# Generalized Poisson Hurdle Model for Count Data and Its Application in Ear Disease

**DOI:** 10.3390/e23091206

**Published:** 2021-09-13

**Authors:** Guoxin Zuo, Kang Fu, Xianhua Dai, Liwei Zhang

**Affiliations:** 1 School of Mathematics and Statistics, Central China Normal University, Wuhan 430079, China; zuogx@mail.ccnu.edu.cn (G.Z.); fukang@mails.ccnu.edu.cn (K.F.); mclimeng@gmail.com (L.Z.); 2 School of Public Administration, Central China Normal University, Wuhan 430079, China; 3 Center for Labor and Social Security Research, Central China Normal University, Wuhan 430079, China

**Keywords:** over-dispersion and under-dispersion, excess of zero, generalized Poisson regression, Hurdle model, generalized Poisson Hurdle model, generalized method of moments

## Abstract

For count data, though a zero-inflated model can work perfectly well with an excess of zeroes and the generalized Poisson model can tackle over- or under-dispersion, most models cannot simultaneously deal with both zero-inflated or zero-deflated data and over- or under-dispersion. Ear diseases are important in healthcare, and falls into this kind of count data. This paper introduces a generalized Poisson Hurdle model that work with count data of both too many/few zeroes and a sample variance not equal to the mean. To estimate parameters, we use the generalized method of moments. In addition, the asymptotic normality and efficiency of these estimators are established. Moreover, this model is applied to ear disease using data gained from the New South Wales Health Research Council in 1990. This model performs better than both the generalized Poisson model and the Hurdle model.

## 1. Introduction

Count data are common in various areas, such as public health, insurance, traffic, and epidemiology. The Poisson model and negative binomial model are usually applied to handle count data. When the sample variance is either larger or smaller than the sample mean, this means that it is over-dispersed or under-dispersed, respectively. The Poisson model and negative binomial model cannot handle over- or under-dispersed count data. Hence, the use of generalized Poisson distribution is proposed for over- or under-dispersed count data [1]. The generalized Poisson regression (GPR) model based on generalized Poisson distribution has been widely studied [2,3]. Additionally, the generalized Poisson Regression model was estimated by the maximum likelihood method and the method of moments [4]. Some measures, such as the Pearson chi-squared test, deviance, the likelihood ratio test, Akaike Information Criteria (AIC), and Bayesian Information Criteria (BIC), have been proposed for testing the goodness of fit of a model. Generalized Poisson Regression performs better than other regression models [5]. Zero inflated Waring distribution (ZIW) has been proposed to solve the problem of overdispersion in the data and the reduction of its mean [6].

For count data with an excess of zeros, zero-inflated regression models, such as zero-inflated Poisson (ZIP) and zero-inflated negative binomial (ZINB), have been proposed [7,8]. The zero-inflated Poisson (ZIP) model deals with count data with an excess of zeros [9], while the zero-inflated negative binomial (ZINB) model deals with over-dispersed count data with excess zeros, for example, the zero-inflated generalized Poisson (ZIGP) model [10,11,12,13,14,15], and the Zero-inflated Bell regression model [16,17]. These models have been used to analyze the relationship between the number of mothers that receive antenatal care visits and other factors, such as maternal education, partner education level, age of mothers, religion of mothers, and wealth index. In particular, these models are compared using AIC and BIC. In addition, the Zero-Inflated Hierarchical Poisson Model has been applied to data with an excess of zeros to analyze maternal mortality data from 2010 to 2013 in health facilities in four regions of Ghana [18].

Although these models deal with zero-inflated data, they cannot simultaneously deal with both zero-deflated and over-/under-dispersed data. An alternative model that accounts for zero-inflated or zero-deflated is the Hurdle model [19]. This model specifies two processes that generate zero counts and positive counts. Feng compared the zero-inflated and Hurdle models to determine the differences and performances of both models in a simulation [20]. The extended negative binomial Hurdle (ENBH) model deals with zero-inflation in addition to under-dispersion in non-zero counts [21]. Bocci et al. generalized the usual Hurdle regression model by specifying a multiple inflated truncated negative binomial distribution for the positive responses and applied it to the tourism behavior of Italian residents [22]. Park and Kim proposed a tree-structured hierarchical model for count data of both excessive zeros and over-dispersion [23]. Hasanah et al. estimated a Hurdle model using the Bayesian method with the non-closed form of posterior distributions by specified non-information priors for parameters [24].

Parameter estimation is important. Estimation methods include the maximum likelihood (ML), the method of moments, the generalized method of moments (GMM), and Bayesian estimation. The GMM method is widely used in parameter estimation due to its good statistical properties. Chen and Cheng proposed a partially linear additive spatial error model (PLASEM), estimated its parameters using GMM, and derived consistency and asymptotic normality for some estimators [25]. Muris investigated missing data using GMM, derived a set of moment conditions, and obtained the efficiency [26]. For count data, Sarvi et al. studied the use of the GEE-based generalized Poisson Regression model for over- and under-dispersed clustered count data with excess zeros [27]. Mahpolah et al. used GMM to estimate a Poisson regression model; the result gained with the use of GMM is better than that gained by the use of ML [28]. Allo et al. estimated the parameters of the generalized Poisson regression model using GMM and applied it to diarrhea in infants in Pasuruan Regency, East Java [29]. Yogita and Kirtee estimated parameters of the zero-inflated model using a probability estimated method based on a moment estimator of the mean parameter [30].

Ear diseases are important in healthcare. For ear disease, Lee et al. used a meta-analysis method for the incidence of ear diseases caused by swimming without ear protection [31]. Sanchez et al. investigated the impact of swimming in water containing saline chloride on the occurrence of ear disease [32]. Subtil et al. investigated whether water precautions reduce the rate of ear diseases [33]. Sanchez et al. measured the impact of 4 weeks of daily swimming on rates of ear discharge with a tympanic membrane perforation and on the middle ear, and found swimming not to be associated with increased risk of ear disease [34].

Count data, for example in ear disease, usually contains too many or too few zeros and exhibits dispersion characteristics meanwhile. This paper proposes the generalized Poisson Hurdle model (GPHR), which simultaneously deals with count data that are both zero-inflated/zero-deflated and over-/under-dispersed, and utilizes GMM to estimate the parameters. Furthermore, the asymptotic normality and efficiency for GMM estimators are established for the GPHR model under certain conditions. As high-order moments are not easy to calculate, the bootstrap method is used to estimate the variance of the GMM estimator in real data. In its application to ear disease using the data gained from New South Wales Health Research Council in 1990, it can be seen that the GMM estimators perform better than the ML estimators of the GPHR model.

The paper is organized as follows. Section 2 introduces the generalized Poisson Hurdle model. Section 3 carries out GMM estimation and establishes its asymptotic normality and efficiency. Section 4 introduces the Nelder mean algorithm in detail. Section 5 discusses the application to real data in ear disease. Section 6 concludes the paper. Technical proofs are given in Appendix A.

## 2. Basic Model

For count data, the generalized Poisson regression (GPR) model performs better with over-dispersion or under-dispersion, while the Hurdle regression model performs better with zero excess data. This paper introduces the generalized Poisson Hurdle regression (GPHR) model to simultaneously deal with both over- or under-dispersion and zero-inflated data.

### 2.1. Generalized Poisson Regression

To handle data with over- or under-dispersion upon generalized Poisson distribution, two main versions of generalized Poisson distribution are used, denoted by GP_1_ and GP_2_.

Suppose that random variable Y follows a GP_1_ distribution [1]. The probability density function of Y is:

$$\mathrm{Pr}\left(Y=y\right)=\{\begin{array}{cc} \frac{\lambda_{1}{\left(\lambda_{1}+y\lambda_{2}\right)}^{y-1}e^{-\left(\lambda_{1}+y\lambda_{2}\right)}}{y!}, & \lambda_{1}+y\lambda_{2}>0,\\ 0, & \lambda_{1}+y\lambda_{2}\;\leq \;0, \end{array}$$

where y=0,1,2,… and $\lambda_{1}$ and $\lambda_{2}$ are unknown parameters.

Another example is generalized Poisson distribution GP_2_ [35]. For random variable Y, the probability density function is:

(1) $$\mathrm{Pr}\left(Y=y\right)={\left[\frac{\mu }{1+\alpha \mu }\right]}^{y}\frac{{\left(1+\alpha y\right)}^{y-1}}{y!}\exp\left[-\frac{\mu \left(1+\alpha y\right)}{1+\alpha \mu }\right],\;y=0,1,2,\ldots$$

where α is a dispersion parameter. If α>0, then the variance is greater than the mean, which is known as overdispersion; if α<0, then the variance is less than the mean, which is known as under-dispersion; and if α=0, then the generalized Poisson distribution degenerates to a Poisson distribution. Hence, the dispersion characteristics of data depends on dispersion parameter α.

As can be seen, GP_1_ is identical to GP_2_, when $\lambda_{1}=\frac{\mu }{1+\alpha \mu },\lambda_{2}=\frac{\alpha \mu }{1+\alpha \mu }$.

If $Y\sim {\mathrm{GP}}_{1}$, the mean and variance are $E\left[Y\right]=\frac{\lambda_{1}}{1-\lambda_{2}}$ and $\mathrm{Var}\left[Y\right]=\frac{E\left[Y\right]}{{\left(1-\lambda_{2}\right)}^{2}}$, respectively.

Similarly, if $Y\sim {\mathrm{GP}}_{2}$, the mean and variance are:

(2) E[Y]=μ

And:

(3) $$\mathrm{Var}\left[Y\right]=\mu {\left(1+\alpha \mu \right)}^{2}$$

The generalized Poisson regression (GPR) model runs as follows:

(4) $$\ln\left(\mu \right)=x^{T}\beta$$

where x is the (p+1)-dimension vector from the predictor variable (with a 1 in the first element) and β is the (p+1)-dimension vector from the regression parameter.

### 2.2. Hurdle Model

The Hurdle regression model, also named the two-part structure, is an effective model in dealing with zero-inflated and zero-deflated data [19]. This model separates the generation of zero data from that of positive data and regards these as two separate processes. The first process judges whether the zero events happen; this is denoted by 0 when zero events happen with probability w and denoted by 1 when zero events do not happen with probability 1−w. When the studied event has happened, we enter into the second process, i.e., how many times the event happens. In this process, the occurrence of the event conforms to, for example, a Poisson distribution, negative binomial distribution, or general Poisson distribution. However, in this process, the event’s value must take a positive value and the events must occur at least once, which is based on the first process. Therefore, the event is conditionally distributed and zero truncated.

This leads us to the Hurdle model:

(5) $$\mathrm{Pr}\left(Y=j\right)=\{\begin{array}{cc} f_{1}\left(0\right)=w, & j=0,\\ \frac{1-f_{1}\left(0\right)}{1-f_{2}\left(0\right)}f_{2}\left(j\right), & j>0, \end{array}$$

where $f_{1}\left(y\right)$ and $f_{2}\left(y\right)$ are the density functions of the first process and second process, respectively. $f_{1}\left(0\right)=w$ is the probability that zero occurs and $\frac{f_{2}\left(y\right)}{1-f_{2}\left(0\right)}$ is the truncated density. The model allows for excess zeros if $f_{1}\left(0\right)>f_{2}\left(0\right)$ and can model too-few zeros if $f_{1}\left(0\right)<f_{2}\left(0\right)$. Obviously, the distribution reduces to the simple f distribution only if $f_{1}=f_{2}=f$.

The moments of the Hurdle model can be easily calculated. The mean is:

(6) $$E\left[Y\right]=\mathrm{Pr}\left[Y>0\right]\cdot E_{Y>0}\left[Y|Y>0\right]=\frac{1-w}{1-f_{2}\left(0\right)}\cdot \upsilon_{2},$$

where $\upsilon_{2}$ is the untruncated mean for the density $f_{2}\left(y\right)$. Similarly, the 2-nd moment is:

$$E\left[Y^{2}\right]=\frac{1-w}{1-f_{2}\left(0\right)}\cdot \sigma_{2}{}^{2}$$

where $\sigma_{2}{}^{2}$ is the untruncated variance for the density $f_{2}\left(y\right)$. Hence, the variance of the Hurdle model is:

(7) $$\mathrm{Var}\left[Y\right]=\frac{1-w}{1-f_{2}\left(0\right)}\cdot \sigma_{2}{}^{2}-{\left[\frac{1-w}{1-f_{2}\left(0\right)}\cdot \upsilon_{2}\right]}^{2}$$

### 2.3. Generalized Poisson Hurdle Regression Model

This subsection combines the advantage of the generalized Poisson regression model for dispersed data and the hurdle model for zero-inflated and zero-deflated data. According to the basic principle of the Hurdle model, if the second process is in a zero-truncated generalized Poisson distribution, then the Generalized Poisson Hurdle Regression model can be proposed as follows:

(8) $$\mathrm{Pr}\left(Y=j\right)=\{\begin{array}{cc} w, & j=0,\\ \left(1-w\right)\frac{g}{1-g\left(0\right)}, & j>0, \end{array}$$

where $g=g\left(y;\mu ,\alpha \right)={\left[\frac{\mu }{1+\alpha \mu }\right]}^{y}\frac{{\left(1+\alpha y\right)}^{y-1}}{y!}\exp\left[-\frac{\mu \left(1+\alpha y\right)}{1+\alpha \mu }\right]$

Using Equations (2), (3), (6) and (7), the mean and variance are:

(9) $$E\left[Y\right]=\frac{1-w}{1-\exp\left(-\frac{\mu }{1+\alpha \mu }\right)}\mu$$

And:

(10) $$\mathrm{Var}\left[Y\right]=\frac{1-w}{1-\exp\left(-\frac{\mu }{1+\alpha \mu }\right)}\cdot \left[\mu {\left(1+\alpha \mu \right)}^{2}+\mu^{2}\right]-{\left[\frac{1-w}{1-\exp\left(-\frac{\mu }{1+\alpha \mu }\right)}\cdot \mu \right]}^{2}$$

Obviously, the GPHR model changes to a simple Poisson Hurdle regression model when α=0. Then, the GPHR model can be followed by Equations (11) and (12).

(11) $$\ln\left(\mu \right)=x^{T}\beta ,$$

(12) $$\log\mathrm{it}\left(w\right)=z^{T}\delta .$$

where x and z are (p+1)-dimension vector as predictor variables (with a 1 in the first element), and β and δ are (p+1)-dimension vector as regression parameters. In general, x and z may be the same in the model, the reason why the estimated coefficients of explanatory variables appear twice in real data analysis.

## 3. Estimation

In this section, we estimate the parameters using the generalized method of moments (GMM) and establish their asymptotically normal properties and efficiencies [36,37].

### 3.1. Parameter Estimation

In simple notation, let $X={\left(x^{T},z^{T}\right)}^{T}$, $\zeta ={\left(\beta^{T},\delta^{T}\right)}^{T}$, where T indicates the transposition for matrix or vector; then, Equations (11) and (12) are simplified to:

(13) $$\log\left(\begin{array}{c} \mu \\ \frac{w}{1-w} \end{array}\right)=\left(\begin{array}{c} x^{T}\beta \\ z^{T}\delta \end{array}\right)=X^{T}\zeta .$$

For the GPHR model, the 1st and 2nd moments can be easily obtained from Equations (9) and (10):

$$E\left[Y\right]=\frac{1-w}{1-\exp\left(-\frac{\mu }{1+\alpha \mu }\right)}\mu =:g_{1}\left(X,\theta \right),\;E\left[Y^{2}\right]=\frac{1-w}{1-\exp\left(-\frac{\mu }{1+\alpha \mu }\right)}\cdot \left[\mu \left(1+\alpha \mu \right)+\mu^{2}\right]=:g_{2}\left(X,\theta \right),$$

where $\theta ={\left(\alpha ,\zeta^{T}\right)}^{T}$ is an unknown parameter. Hence, when X is non-random, the moment condition for the GPHR model can be followed by Equation (14):

(14) $$h\left(Y,X,\theta \right)=\left[\begin{array}{c} X\left(Y-g_{1}\left(X,\theta \right)\right)\\ X\left(Y^{2}-g_{2}\left(X,\theta \right)\right) \end{array}\right].$$

As can easily be seen, $E\left[h\left(Y,X,\theta_{0}\right)\right]=0$, where $\theta_{0}$ is the vector of the true parameters. Using Equation (14), the sample moment condition can be obtained.

(15) $$h_{n}\left(Y,X,\theta \right)=\frac{1}{n}\overset{n}{\underset{i=1}{\sum }}h\left(Y_{i},X_{i},\theta \right).$$

Then, we can obtain the objective function:

(16) $$Q_{n}\left(Y,X,\theta \right)={\left[\frac{1}{n}\overset{n}{\underset{i=1}{\sum }}h\left(Y_{i},X_{i},\theta \right)\right]}^{T}W\left(\theta \right)\left[\frac{1}{n}\overset{n}{\underset{i=1}{\sum }}h\left(Y_{i},X_{i},\theta \right)\right],$$

where W(θ) is a weight matrix. There are several common forms for the weight matrix. for example, an identity matrix and the covariance matrix of the estimating equation vector. Without ambiguity, let $Q_{n}\left(\theta \right)=Q_{n}\left(Y,X,\theta \right)$,$h_{n}\left(\theta \right)=h_{n}\left(Y,X,\theta \right)$ and W=W(θ). Let $\hat{\theta }$ be a minimizer of Equation (16); then, $\hat{\theta }$ is called the GMM estimator.

### 3.2. Asymptotic Property and Efficiency

In this subsection, we investigate the efficiency and asymptotic normality of the GMM estimator. Let the true value of the parameter $\theta_{0}\;\in \;\Theta ,$ where Θ is a compact set. For a matrix A, let $\|A\|={\left(\sum a_{ij}{}^{2}\right)}^{\frac{1}{2}}$. Some assumptions are required.

**Assumption** **1.**

*(i)*  *The covariate* X *is a non-random variable;*
*(ii)*  *The weight matrix* W *is positive definite matrix;*
*(iii)*  $WE\left[h\left(Y,X,\theta \right)\right]=0\Leftrightarrow \theta =\theta_{0}$.

Assumption 1 ensures the identification of the GMM estimator by Lemma 1 and this is a condition for the existence of the GMM estimator [38].

**Lemma** **1:** If W is a positive semi-definite matrix, let $h_{0}\left(\theta \right)=E\left[h\left(Y,X,\theta \right)\right]$. Assume that $h_{0}\left(\theta_{0}\right)=0$ and $h_{0}\left(\theta \right)\neq 0$ for $\theta \neq \theta_{0}$. Then, $Q_{0}\left(\theta \right)={\left[h_{0}\left(\theta \right)\right]}^{T}W\left[h_{0}\left(\theta \right)\right]$ has a unique minimum value at $\theta =\theta_{0}$.

To rigorously establish the consistency and asymptotic normality of the estimator, the following regular assumptions are required.

**Assumption** **2.**

(i)
$g_{1}\left(X,\theta \right)$ *and* $g_{2}\left(X,\theta \right)$ *are continuous functions in* θ∈Θ *with probability one;*
(ii) $g_{1}\left(X,\theta \right)$*and*$g_{2}\left(X,\theta \right)$*are continuously differentiable in a neighborhood*$N\left(\theta_{0}\right)$*of*$\theta_{0}$.

As can easily be seen, Assumption 2 is equivalent to the statement that h(Y,X,θ) is continuously differentiable in neighborhood $N\left(\theta_{0}\right)$ of $\theta_{0}$.

**Assumption** **3.** *There exist* $C_{1}>0$ *and* $C_{2}>0$ *such that*

$$\underset{\theta \in \Theta }{sup}||g_{1}\left(X,\theta \right)||<C_{1},\underset{\theta \in \Theta }{sup}||g_{2}\left(X,\theta \right)||<C_{2}.$$

**Assumptio** **4.** $Var\left[Y^{k}\right],k=1,2$ *are bounded away from both zero and infinity uniformly.*

The above assumptions ensure the consistency of the estimator. Hence, the following theorem holds.

**Theorem** **1.** *Let observed data*$\left\{\left(Y_{i},X_{i}\right),i=1,2,\ldots ,n\right\}$*be i.i.d. If assumptions 1-4 hold, then*

$$\hat{\theta }\overset{P}{\to }\theta_{0},\;$$

*where* $\hat{\theta }=\arg\underset{\theta }{\min}Q_{n}\left(\theta \right)$.

To establish the asymptotic normality of the proposed estimator, some strict conditions are required on the moment and on the parameter space Θ.

**Assumption** **5.** $\sqrt{n}h_{n}\left(\theta_{0}\right)\overset{d}{\to }N\left(0,\Sigma \right)$ and $E\left[||h_{n}{\left(\theta_{0}\right)||}^{2}\right]<\infty$.

**Assumption** **6.** *There exist* $G\left(\theta \right)=E\left[𝛻h_{n}\left(\theta \right)\right]$ *and* $G\equiv G\left(\theta_{0}\right)$ *, such that* $G^{T}WG$ *is nonsingular matrix.*

Assumptions 5 and 6 are required for the asymptotic variance and its asymptotic normality. Similar to the conditions of theorem 3.4 in [38], asymptotic normality holds.

**Theorem** **2 (Asymptotic normality).** *Let the observed data*$\left\{\left(Y_{i},X_{i}\right),i=1,2,\ldots ,n\right\}$*be i.i.d. If assumptions 1-6 hold, then:*

$$\sqrt{n}\left(\hat{\theta }-\theta_{0}\right)\overset{d}{\to }N\left(0,\Gamma \right),\;$$

*where*$\Gamma ={\left(G^{T}WG\right)}^{-1}G^{T}W\Sigma WG{\left(G^{T}WG\right)}^{-1}$.

As can be seen from Theorem 2, the asymptotic variance can be simplified to ${\left(G^{T}\Sigma^{-1}G\right)}^{-1}$ when $W=\Sigma^{-1}$. In some cases, the efficiency of the estimator is established—that is, an estimator has minimum variance. For an unbiased estimator, an unbiased estimator (MVUE) with a minimum variance can be easily defined. In fact, if the estimator is unbiased, then it is asymptotically unbiased; hence, we concentrate on asymptotically unbiased estimators with minimum variance. For matrix P and Q, P>Q and P ≥ Q, where P−Q is a positive definite matrix and a positive semi-definite matrix. As a special case, the following theorem holds:

**Theorem** **3.** *Let the conditions of Theorem 2 hold. If*

$$\Sigma =E\left[h{\left(Y,X,\theta_{0}\right)}^{T}h\left(Y,X,\theta_{0}\right)\right]\;$$

*is nonsingular matrix and* $W=\Sigma^{-1}$ *, then:*

$$\sqrt{n}\left(\hat{\theta }-\theta_{0}\right)\overset{d}{\to }N\left(0,{\left(G^{T}\Sigma^{-1}G\right)}^{-1}\right).\;$$

*Hence, the* $\hat{\theta }$ *is the estimator of minimum variance.*

As can be seen from Theorem 3, the GMM estimator is most efficient when the weight matrix is identical to the inverse of the covariance of estimating function h(Y,X,θ) in all families of GMM.

## 4. Algorithm

Indeed, it is difficult to calculate the first derivative (16). However, GMM estimation can be calculated by the Nelder–Mead algorithm [39], which is widely known as the Nelder–Mead simplex algorithm, to find a minimum of the function of several variables. In particular, for complex functions, this algorithm is a good choice, since it does not require differentiation.

The Nelder–Mead algorithm runs as follows.

1. Choose m+1 point, $\theta_{1},\theta_{2},\ldots ,\theta_{m},\theta_{m+1}$, $\theta_{j}\in R^{m}$ and $Q_{n}\left(\theta_{1}\right)\leq \ldots \leq Q_{n}\left(\theta_{m+1}\right)$.
2. Calculate $\overset{-}{\theta }=\frac{1}{m}\overset{m}{\underset{i=1}{\sum }}\theta_{i}$.
3. Compute $\theta_{k}=\left(1+\eta \right)\overset{-}{\theta }-\eta \theta_{m+1}$, where η>0 is a reflection coefficient. If $Q_{n}\left(\theta_{1}\right)\;\leq \;Q_{n}\left(\theta_{k}\right)\;\leq \;Q_{n}\left(\theta_{m}\right)$, then $\theta_{k}\to \theta_{m+1}$, else go as follows:
  - If $Q_{n}\left(\theta_{k}\right)\;\leq \;Q_{n}\left(\theta_{1}\right)$, then calculate $\theta^{*}=\left(1-\gamma \right)\overset{-}{\theta }+\gamma \theta_{k}$, where γ>1 is an expansion coefficient. Next, If $Q_{n}\left(\theta^{*}\right)\;\leq \;Q_{n}\left(\theta_{k}\right)$, then $\theta^{*}\to \theta_{m+1}$, else $\theta_{k}\to \theta_{m+1}$.
  - If $Q_{n}\left(\theta_{m}\right)\;\leq \;Q_{n}\left(\theta_{k}\right)\;\leq \;Q_{n}\left(\theta_{m+1}\right)$, then calculate $\theta^{**}=\left(1-\xi \right)\overset{-}{\theta }+\xi \theta_{k}$, where 0<ξ<1 is a contraction coefficient. Next, if $Q_{n}\left(\theta^{**}\right)\;\leq \;Q_{n}\left(\theta_{k}\right)$, then $\theta^{**}\to \theta_{m+1}$, else go to Step 4.
  - If $Q_{n}\left(\theta_{k}\right)\;\geq \;Q_{n}\left(\theta_{m+1}\right)$, then calculate $\theta^{***}=\left(1+\xi \right)\overset{-}{\theta }-\xi \theta_{k}$. Next, if $Q_{n}\left(\theta^{***}\right)\;\leq \;Q_{n}\left(\theta_{m+1}\right)$, then $\theta^{***}\to \theta_{m+1}$, else go to Step 4.
4. $\left(1-\kappa \right)\theta_{1}+\kappa \theta_{i}\to \theta_{i}$, where 0<κ<1 and 2 ≤ i ≤ m+1.

In the algorithm above, symbol A→B indicates replacing B with A. For the Nelder–Mead algorithm, the standard settings of η,γ,ξ,κ are $\eta =1,\;\gamma =2,\;\xi =\frac{1}{2},\;\kappa =\frac{1}{2}$. Usually, the iteration stops when ${\left\{m^{-1}\overset{m}{\underset{i=1}{\sum }}\left[Q_{n}\left(\theta_{i}\right)-{\overset{-}{Q}}_{n}\left(\theta \right)\right]\right\}}^{\frac{1}{2}}<\varepsilon$, or when the number of iterations reaches a fixed value.

In practice, the Nelder–Mead algorithm is widely used to optimize target functions, since it simply and easily achieves its minimization. It does not require continuous or differentiable target functions. This algorithm is significantly improved in the first few iterations and quickly provides satisfactory results. However, there are two disadvantages of the Nelder–Mead algorithm. The algorithm is very sensitive to the initial values. Additionally, the convergence of the algorithm is difficult to guarantee globally, even for smooth and well-behaved functions. See, for example, for special conditions [40,41,42,43]. The Nelder–Mead algorithm is modified to improve the worst-case performance of the algorithm in terms of convergence, but retains some or most of its efficiency in best-case scenarios [44,45].

## 5. Real Data Analysis

As a healthy sport, swimming can improve human energy metabolism and maintain respiratory health. However, frequent swimming may lead to excessive moisture in the ear and inflammation of the external auditory canal. Moisture can cause ear eczema. Skin damage caused by repeated scratching of eczema can make bacteria or fungi invade the ear canal tissue and cause infection. However, swimming in bacterially-contaminated water is a common cause of swimming ear disease. Therefore, in health care, it is significant in practice to explore the relationship between the frequency of ear disease and other factors such as swimming. On the other hand, people who often swim may not be infected with ear diseases.

In this section, we analyze real data in ear disease using the method proposed above, and compare results between different methods.

### 5.1. Data Description

**Figure 1:**
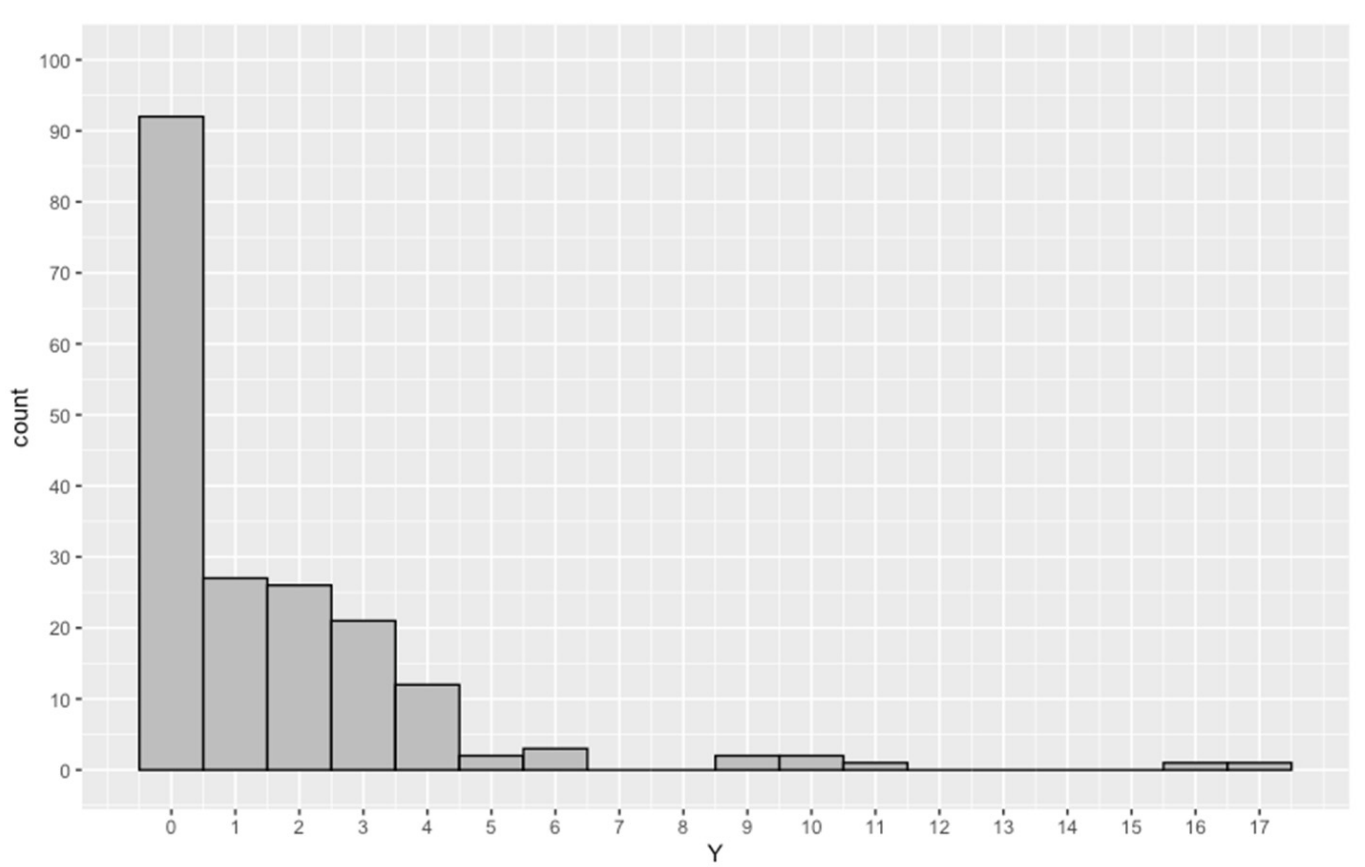
Histogram of number of ear diseases.

The real data in this application relate to the incidence of ear diseases and come from the investigation carried out by the New South Wales Health Research Council in 1990. The data gathered include the frequency of swimming, the place of swimming, and the frequency of ear diseases, from a total of 190 observation data points. The number of ear diseases is recorded by a type of count data—i.e., how many times the self-diagnosis of ear infection has occurred. The frequency of swimming is a categorical variable—i.e., how often the swimmer swims in the ocean—and takes two values: “Often” or “Occas”; quantitative values of “Often” and “Occas” are 1 and 2, respectively. The place of swimming is also a categorical variable, relating to the usual swimming location, and takes the values “Beach” or “Non-beach”; quantitative values of “Beach” and “Nonbeach” are 1 and 4, respectively. In this paper, we use the frequency of swimming and place of swimming as explanatory variable x in our model and the number of ear diseases as response variable Y. Figure 1 is a histogram of the frequency distribution of ear disease, where the value interval of the number of ear disease is (0, 17) and 92 cases occurred in 0, accounting for 48.4% of the total; 27 occurred in 1, accounting for 14.2% of the total; 26 occurred in 2, accounting for 13.7% of the total; 21 occurred in 3, accounting for 11.1% of the total; and 12 occurred in 4, accounting for 6.3% of the total. As the number of occurrences increases, the proportion of the number of ear disease becomes smaller and smaller. As shown in Figure 1, the number of cases of ear disease has a larger probability accumulation at zero. Therefore, if there is zero excess in this set of data, the use of the Hurdle model in this paper may provide a better fit with the data. Information relating to the number characters for variable Y is shown in Table 1. As can be seen in Table 1, the sample data contain more zeros, the expected number of occurrences is 1.6, and the variance is 6.5. The variance is larger than expected—that is, the sample data suffer from over-dispersion. Therefore, the generalized Poisson Hurdle regression model is applied in this real data.

### 5.2. Empirical Analysis

In this subsection, we utilize the generalized Poisson (GP) regression model, the Poisson Hurdle (PH) regression model, and the GPHR model for ear disease. We estimate parameters using the ML method and GMM. For GMM, the initial value used is ML estimation. The Akaike Information Criteria (AIC) are introduced to compare the fitting effects of these three models, where the form of the AIC statistics is AIC =−2l+2p, l is the log-likelihood value, and p is the number of free parameters. In general, the smaller the AIC values, the better the fitting effect of the model.

**Figure 2:**
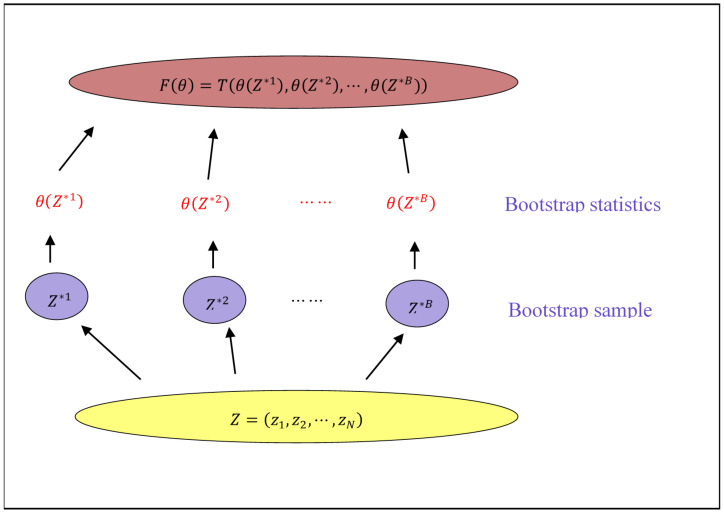
Schematic of the bootstrap process.

In order to make statistical inferences, the variance of the regression coefficients is estimated. It is natural to attempt to derive a consistent estimator, but as seen from the proof of the theorem, such an estimator is too complex and not practical. Hence, we use the bootstrap method to estimate variance. As shown in Figure 2, the basic schematic of bootstrapping proceeds. We denote the training set by $Z=\left(z_{1},z_{2},\ldots ,z_{N}\right)$, randomly draw data sets with replacements from training set Z, and name these bootstrap data sets. The size of the bootstrap data sets may be not equal to that of the original training set. We repeat B times and produce B bootstrap data sets. Then, we refit the model to each of the bootstrap data sets and examine the behavior of the fits over the i-th bootstrap data set.

In Figure 2, θ(Z) represents any statistics computed from the data set Z. Using bootstrap sampling, we can estimate any aspect F(θ) of the distribution of θ(Z), for example, its variance:

$$\hat{\mathrm{Var}}\left[\theta \left(Z\right)\right]=\frac{1}{B-1}\overset{B}{\underset{i=1}{\sum }}{\left[\theta \left(Z^{*i}\right)-{\overset{-}{S}}^{*}\right]}^{2},\;$$

where ${\overset{-}{S}}^{*}=\frac{1}{B}\overset{B}{\underset{i=1}{\sum }}\theta \left(Z^{*i}\right)$. For the bootstrap method used, see, for example, Efron and Tibshirani [46]. In this paper, all programs and algorithms are calculated by R in version 3.6.3 and Rstudio in version 1.2.5042.

Table 2 shows the results for three models. For the generalized Poisson Hurdle regression model and the Poisson hurdle model, each model includes two parameters related to the explanatory variables, and the coefficients of explanatory variable are estimated twice, i.e., intercept, frequency and place appear twice in the results. The Wald confidence intervals at the significance of 5% and p-values are also shown in Table 2. To estimate the variance of GMM, bootstrap methods are used where B=50 times. The GMM estimation and ML estimation produce similar results for the three models. The PH model obtains the worst results and the results of the AIC are relatively large, which may be due to the over-dispersion. The GP regression model and the GPHR model both have over-dispersed characteristics, meaning that the fitting effect is improved. On the other hand, the GPHR model improves the results in the case of zero excess, as can be seen from the AIC value, compared with the GP regression model. The variances of the GMM estimator are less than the variances of the ML estimator for three models. Additionally, it can be seen that people who often swim are more likely to suffer from ear disease. Furthermore, it is evident that swimming at the beach has a negative impact on ear disease. Under the null hypothesis, this statistic is asymptotically normally distributed. At the 5% significance level, the GPHR model fits the data for ear disease better than both the GP regression model and the PH regression model.

## 6. Conclusions

This paper introduces the GPHR model for count data. It combines the generalized Poisson Regression (GPR) model with the Hurdle Regression model. Compared with other models, such as the PH regression model and the GP regression model, the GPHR model can simultaneously deal with both zero-inflated or zero-deflated data and over- or under-dispersion in count data. GMM is used to estimate the parameters of the models. In some cases, the GMM performs better than ML. Since it is difficult to calculate the first derivative of the objective function, the Nelder–Mead method is used to estimate the parameters.

Ear diseases are important in healthcare. The proposed model is applied to real data on ear diseases, which is another purpose of this paper. Compared with other zero-inflated models, the GPHR model has a minimum AIC value. Given a significant level (5%), the Wald test is used to measure the significance of the factors incorporated in each model. As shown, the GPHR model fits well with the real data. In fact, one count data is not enough to validate advantages and disadvantages of the purposed model for both zero-inflated or zero-deflated data and over- or under-dispersion. Although this model performs well in the data for ear disease, other count data are better at checking this model. Indeed, we check consumer health information for medical treatment, and this model also performs well. We do not report in detail, since this paper aimed to purpose the GPHR model for count data of both zero-inflated or zero-deflated data and over- or under-dispersion, and apply this to ear disease data.

In this paper, we only studied data that were zero-inflated. The model may be extended for other types of count data, such as zero-deflated data. In addition, for the first process of the Hurdle model, the occurrence probability w is used. In fact, this may be extended to the Poisson distribution at zero when the first process occurs, with the same parameter μ used as one in the second process. For the second process of the Hurdle model, this may be extended to the case of truncation.

## Figures and Tables

**Table 1:** Some number characters for ear disease (NED).

Variable	Min.	1st Qu.	Med.	Mean	3rd Qu.	Max.	Var.
NED	0.0	0.0	1.0	1.6	2.0	17.0	6.5

Note: Number characters of the number of ear disease (NED) include the minimum value (Min.), first quartile (1st Qu.), median (Med.), third quartile (3rd Qu.), maximum value (Max.) and variance (Var.).

**Table 2:** Estimation results for real data.

Variables	GPHR				PH				GP			
	MLE		GMM		MLE		GMM		MLE		GMM	
	Coefficient	SE	Coefficient	SE	Coefficient	SE	Coefficient	SE	Coefficient	SE	Coefficient	SE
DIS	0.28 (0.1, 0.4)	0.09 (0.002)	0.17 (0.07, 0.3)	0.05 (<0.001)	−	−	−	−	0.59 (0.4, 0.8)	0.10 (<0.001)	0.52 (0.4, 0.6)	0.06 (<0.001)
INT1	−0.48 (−1.8, 0.8)	0.66 (0.474)	−0.42 (−0.6, −0.3)	0.07 (<0.001)	−0.08 (−0.8, 0.7)	0.38 (0.837)	0.09 (−0.2, −0.01)	0.04 (0.002)	−1.56 (−2.8, −0.3)	0.64 (0.016)	−1.43 (−1.5, −1.3)	0.06 (<0.001)
FRE1	0.63 (0.1, 1.1)	0.26 (0.016)	0.67 (0.5, 0.8)	0.08 (<0.001)	0.55 (0.2, 0.9)	0.16 (<0.001)	0.41 (0.3, 0.6)	0.08 (<0.001)	0.75 (0.2, 1.3)	0.26 (0.004)	0.71 (0.6, 0.8)	0.06 (<0.001)
PLA1	0.07 (−0.1, 0.2)	0.10 (0.513)	0.11 (0, 0.2)	0.06 (0.06)	0.06 (−0.05, 0.2)	0.06 (<0.001)	0.03 (−0.1, 0)	0.05 (0.543)	0.23 (0.03, 0.4)	0.10 (0.017)	0.20 (0.1, 0.3)	0.04 (<0.001)
INT2	2.29 (0.6, 4.0)	0.85 (0.007)	2.35 (2.2, 2.4)	0.07 (<0.001)	−2.29 (−3.9, −0.6)	0.85 (0.007)	2.08 (−2.2, −2.0)	0.05 (<0.001)	−	−	−	−
FRE2	−0.71 (−1.3, −0.02)	0.35 (0.040)	−0.72 (−1, −0.4)	0.14 (<0.001)	0.71 (0.02, 1.3)	0.35 (0.004)	0.83 (0.7, 0.9)	0.05 (<0.001)	−	−	−	−
PLA2	−0.37 (−0.6, −0.1)	0.13 (0.004)	−0.29 (−0.4, −0.1)	0.07 (<0.001)	0.37 (0.1, 0.6)	0.13 (0.004)	0.57 (0.5, 0.6)	0.03 (<0.001)	−	−	−	−
AIC	639.90				745.64				643.31			

Note: DIS, INT, FRE and PLA are abbreviations for dispersion parameter α, model intercept, frequency, and place, respectively; generalized Poisson Hurdle regression model (GPHR), Poisson hurdle regression model (PH), and generalized Poisson regression model (GP) for ear disease data. The coefficients are estimated by maximum likelihood (MLE) method and generalized method of moment (GMM), and standard error (SE) is calculated by Fisher information matrix and bootstrap method, respectively. The numbers in the parentheses below coefficient and standard error (SE) are Wald confidence intervals and *p*-values, respectively. For GPHR and PH model, the coefficients of INT1, FRE1 and PLA1 are count model coefficients β, while the coefficients of INT2, FRE2 and PLA2 are zero Hurdle model coefficients δ.

## Data Availability

Data used in this paper can be found from https://search.r-project.org/CRAN/refmans/GLMsData/html/earinf.html.

## Appendix A

To prove the main theorems, we need the following lemma:

**Lemma** **A1.** [47]: *Let observed data* $\left\{X_{i},i=1,2,\ldots ,n\right\}$ *be i.i.d random variable, and the parametric space compact set. Function* G(X,θ) *is continuous for* θ∈Θ *with probability 1. If there exists a function* B(X) *such that* G(X,θ)≤B(X) *for all* θ∈Θ*, and* E[B(X)]<∞*, then* E[G(X,θ)] *is continuous and*

$$\underset{\theta \in \Theta }{\sup}||\frac{1}{n}\overset{n}{\underset{i=1}{\sum }}G\left(X_{i},\theta \right)-E\left[G\left(X,\theta \right)\right]||\overset{P}{\to }0.$$

**Proof of Theorem** **1.** Using lemma 1 and assumption 1, we can determine that $Q_{0}\left(\theta \right)$ has a unique minimum value $\theta_{0}$. Next, we prove that the $Q_{n}\left(\theta \right)$ uniformly converges to $Q_{0}\left(\theta \right)$ with a high probability.

Based on assumptions 2 and 3:

$$||h\left(Y,X,\theta \right)||\leq ||X^{T}X||\cdot \left(||Y-g_{1}\left(X,\theta \right)||+||Y^{2}-g_{2}\left(X,\theta \right)||\right).$$

i.e., $E\underset{\theta \in \Theta }{\sup}||h\left(Y,X,\theta \right)||<\infty$. Then, based on Lemma A1, $h_{0}\left(\theta \right)$ is continuous and:

$$\underset{\theta \in \Theta }{\sup}||h_{n}\left(\theta \right)-h_{0}\left(\theta \right)||\overset{P}{\to }0.$$

In addition, $Q_{0}\left(\theta \right)$ is continuous.

Due to triangle inequality and Cauchy–Schwartz inequality:

$$\begin{array}{l} \left|Q_{n}\left(\theta \right)-Q_{0}\left(\theta \right)\right|\;\\ =\left|{\left[h_{n}\left(\theta \right)\right]}^{T}W\left[h_{n}\left(\theta \right)\right]-{\left[h_{0}\left(\theta \right)\right]}^{T}W\left[h_{0}\left(\theta \right)\right]\right|\;\\ \leq \;\left|{\left[h_{n}\left(\theta \right)-h_{0}\left(\theta \right)\right]}^{T}W\left[h_{n}\left(\theta \right)-h_{0}\left(\theta \right)\right]-{\left[h_{n}\left(\theta \right)\right]}^{T}\left(W+W^{T}\right)\left[h_{n}\left(\theta \right)-h_{0}\left(\theta \right)\right]\right|\;\\ \leq \;||h_{n}\left(\theta \right)-h_{0}{\left(\theta \right)||}^{2}\cdot ||W||+2||W||\cdot ||h_{n}\left(\theta \right)||\cdot ||h_{n}\left(\theta \right)-h_{0}\left(\theta \right)||, \end{array}$$

Then $\underset{\theta \in \Theta }{\sup}\left|Q_{n}\left(\theta \right)-Q_{0}\left(\theta \right)\right|\overset{P}{\to }0$, i.e., $Q_{n}\left(\theta \right)$ uniformly converges to $Q_{0}\left(\theta \right)$ with probability. Recall that Θ is a compact set; for any given ε>0:

$$Q_{n}\left(\hat{\theta }\right)<Q_{n}\left(\theta_{0}\right)+\frac{\varepsilon }{3},Q_{0}\left(\hat{\theta }\right)<Q_{0}\left(\hat{\theta }\right)+\frac{\varepsilon }{3},$$

And:

$$Q_{n}\left(\theta_{0}\right)<Q_{0}\left(\theta_{0}\right)+\frac{\varepsilon }{3}$$

With probability of 1. Hence:

$$Q_{0}\left(\hat{\theta }\right)<Q_{n}\left(\hat{\theta }\right)+\frac{\varepsilon }{3}<Q_{n}\left(\theta_{0}\right)+\frac{2\varepsilon }{3}<Q_{0}\left(\theta_{0}\right)+\varepsilon .$$

Thus, with a probability of 1:

$$Q_{0}\left(\hat{\theta }\right)<Q_{0}\left(\theta_{0}\right)+\varepsilon .$$

Set ℳ as any open set, such that ℳ⊆Θ and $\theta_{0}\in ℳ$. Additionally, $\Theta \cap {ℳ}^{c}$ is a compact set. Thus:

$$Q_{0}\left(\tilde{\theta }\right)\;\equiv \underset{\theta \in \Theta \cap {ℳ}^{c}}{\inf}Q_{0}\left(\theta \right)>Q_{0}\left(\theta_{0}\right),$$

where $\tilde{\theta }\in \Theta \cap {ℳ}^{c}$. Let $\varepsilon =Q_{0}\left(\tilde{\theta }\right)-Q_{0}\left(\theta_{0}\right)$, then:

$$Q_{0}\left(\hat{\theta }\right)<Q_{0}\left(\tilde{\theta }\right).$$

Additionally, $\hat{\theta }\in ℳ$ with a probability of 1. Due to the arbitrariness of ℳ:

$$\hat{\theta }\overset{P}{\to }\theta_{0}.\;□$$

**Proof of Theorem** **2.** Based on assumption 2, h(Y,X,θ) and $h_{n}\left(\theta \right)$ are continuous in $N\left(\theta_{0}\right)$. Under the condition of the extreme minimum value:

(A1) $$𝛻Q_{n}\left(\theta \right)\;=\;2{\left[{𝛻}_{\theta }h_{n}\left(\theta \right)\right]}^{T}Wh_{n}\left(\theta \right)\;=\;0$$

With a probability of 1. Denote $G_{n}\left(\theta \right)={𝛻}_{\theta }h_{n}\left(\theta \right)$. Applying Taylor expansion:

(A2) $$h_{n}\left(\hat{\theta }\right)\;=\;h_{n}\left(\theta_{0}\right)+G_{n}\left(\tilde{\theta }\right)\left(\hat{\theta }\;-\;\theta_{0}\right),$$

where $\tilde{\theta }$ lies between $\theta_{0}$ and $\hat{\theta }$. By multiplying $G_{n}{\left(\hat{\theta }\right)}^{T}W$ (A2) from the left:

$$G_{n}{\left(\hat{\theta }\right)}^{T}Wh_{n}\left(\hat{\theta }\right)\;=\;G_{n}{\left(\hat{\theta }\right)}^{T}Wh_{n}\left(\theta_{0}\right)\;+\;G_{n}{\left(\hat{\theta }\right)}^{T}WG_{n}\left(\tilde{\theta }\right)\left(\hat{\theta \;}-\;\theta_{0}\right).$$

Based on (A1):

$$\sqrt{n}\left(\hat{\theta }-\theta_{0}\right)\;=-\sqrt{n}{\left[G_{n}{\left(\hat{\theta }\right)}^{T}WG_{n}\left(\tilde{\theta }\right)\right]}^{-1}G_{n}{\left(\hat{\theta }\right)}^{T}Wh_{n}\left(\theta_{0}\right).$$

Based on assumptions 1–4, Theorem 1 holds—i.e., $\hat{\theta }\overset{P}{\to }\theta_{0}$. In addition, since X is nonrandom:

$$G_{n}\left(\hat{\theta }\right)\overset{P}{\to }G,\;\mathrm{and}\;G_{n}\left(\tilde{\theta }\right)\overset{P}{\to }G.$$

Hence:

$$-{\left[G_{n}{\left(\hat{\theta }\right)}^{T}WG_{n}\left(\tilde{\theta }\right)\right]}^{-1}G_{n}{\left(\hat{\theta }\right)}^{T}W\overset{P}{\to }-{\left[G^{T}WG\right]}^{-1}G^{T}W.$$

Based on the Slutsky theorem and assumption 5, the asymptotic variance can be established. Thus, theorem 2 holds. □

**Proof of Theorem** **3.** Based on Theorem 2, the covariance matrix of the estimator is ${\left(G^{T}\Sigma^{-1}G\right)}^{-1}$ when $W=\Sigma^{-1}$. Hence, $\sqrt{n}\left(\hat{\theta }-\theta_{0}\right)\overset{d}{\to }N\left(0,{\left(G^{T}\Sigma^{-1}G\right)}^{-1}\right).$

Next, the estimator has minimum variance in this case. Indeed, let $\Sigma =\;E\left[h{\left(Y,X,\theta_{0}\right)}^{T}h\left(Y,X,\theta_{0}\right)\right]$, $\eta =G^{T}Wh\left(Y,X,\theta_{0}\right)$, $\overset{-}{\eta }=G^{T}\Sigma^{-1}h\left(Y,X,\theta_{0}\right)$. Thus:

$$G^{T}WG=E\left[\eta {\overset{-}{\eta }}^{T}\right],\;G^{T}\Sigma^{-1}G=E\left[\overset{-}{\eta }{\overset{-}{\eta }}^{T}\right],\;\mathrm{and}\;G^{T}W\Sigma WG=E\left[\eta \eta^{T}\right].$$

By some simple algebraic manipulation:

$${\left(G^{T}WG\right)}^{-1}G^{T}W\Sigma WG{\left(G^{T}WG\right)}^{-1}-{\left(G^{T}\Sigma^{-1}G\right)}^{-1}={\left(G^{T}WG\right)}^{-1}E\left\{\eta -E\left[\eta {\overset{-}{\eta }}^{T}\right]{\left(E\left[\overset{-}{\eta }{\overset{-}{\eta }}^{T}\right]\right)}^{-1}\overset{-}{\eta }\right\}{\left(G^{T}WG\right)}^{-1}.$$

Since $E\left\{\eta -E\left[\eta {\overset{-}{\eta }}^{T}\right]{\left(E\left[\overset{-}{\eta }{\overset{-}{\eta }}^{T}\right]\right)}^{-1}\overset{-}{\eta }\right\}$ is a positive semi-definite matrix:

$${\left(G^{T}WG\right)}^{-1}G^{T}W\Sigma WG{\left(G^{T}WG\right)}^{-1}-{\left(G^{T}\Sigma^{-1}G\right)}^{-1}$$

This is also a positive semi-definite matrix—i.e.,:

$${\left(G^{T}\Sigma^{-1}G\right)}^{-1}\;\leq \;{\left(G^{T}WG\right)}^{-1}G^{T}W\Sigma WG{\left(G^{T}WG\right)}^{-1}.$$

Hence, $\hat{\theta }$ is the estimator of minimum variance. □
